# Long-Term Exposure to High Altitude Affects Response Inhibition in the Conflict-monitoring Stage

**DOI:** 10.1038/srep13701

**Published:** 2015-09-01

**Authors:** Hailin Ma, Yan Wang, Jianhui Wu, Ping Luo, Buxin Han

**Affiliations:** 1 Key Laboratory of Mental Health, Institute of Psychology, Chinese Academy of Sciences, Beijing, China; 2 University of Chinese Academy of Sciences, Beijing, China; 3 Institute of Education and Psychology, Tibet University, Tibet, China; 4 Key Laboratory of Behavioral Science, Institute of Psychology, Chinese Academy of Sciences, Beijing, China

## Abstract

To investigate the effects of high-altitude exposure on response inhibition, event-related potential (ERP) components N2 and P3 were measured in Go/NoGo task. The participants included an ‘immigrant’ high-altitude group (who had lived at high altitude for three years but born at low altitude) and a low-altitude group (living in low altitude only). Although the behavioural data showed no significant differences between the two groups, a delayed latency of NoGo-N2 was found in the high-altitude group compared to the low-altitude group. Moreover, larger N2 and smaller P3 amplitudes were found in the high-altitude group compared to the low-altitude group, for both the Go and NoGo conditions. These findings suggest that high-altitude exposure affects response inhibition with regard to processing speed during the conflict monitoring stage. In addition, high altitude generally increases the neural activity in the matching step of information processing and attentional resources. These results may provide some insights into the neurocognitive basis of the effects on high-altitude exposure on response inhibition.

As of 2006, approximately 12 million people resided in the Qinghai-Tibetan Plateau^1^. In recent years, more and more people who were born and raised in low-altitude areas are travelling or working in Tibet (average altitude over 4000 m). Living at such a high altitude, the largest and most important impact is hypoxia, which occurs because of a reduction of oxygen in the air and which affects cognition.

Long-term exposure to high altitude with hypoxia may lead to impairment on response inhibition. Previous studies have provided neuroimaging evidence of the impact of high-altitude exposure on the human brain. Evidence from functional magnetic resonance imaging (fMRI) studies has shown that activation of the prefrontal cortex (PFC) and the anterior cingulate cortex (ACC) are associated with response inhibition^2^,^3^. A meta-analysis of Go/NoGo tasks reported that a mainly right-lateralized network is associated with response inhibition, including the right middle/inferior frontal gyrus, right inferior parietal regions, and medial frontal gyrus^4^. Moreover, structural modification of the inferior and middle frontal gyrus and ACC in a group of people born and raised at high altitude was found in chronic hypoxia research using magnetic resonance imaging (MRI)^5^,^6^. Based on the influence of altitude on the ACC and middle frontal gyrus, the response inhibition associated cortex could be affected, indicating that the response inhibition is influenced by high-altitude exposure. Response inhibition is an essential component of cognitive ability and an important aspect of executive function. However, there is no direct evidence on whether long-term exposure to high altitude will influence the response inhibition.

Compared with fMRI and MRI techniques, event-related potentials (ERPs) have high temporal resolution and can provide more insight into the time course of brain processes, making it possible to determine which stage of response inhibition processing is affected by high-altitude exposure^7^. Two ERP components correlate with response inhibition during performance of the Go/NoGo paradigm^8^,^9^. First, the frontocentral N2, related to response inhibition, was located in the right lateral orbitofrontal, right inferior frontal, middle frontal, and anterior cingulate cortex^10^,^11^. It is a negative potential with a latency range of 200–300 ms, the NoGo-N2 amplitude increased in the NoGo compared to the Go trial, reflecting a conflict-monitoring process in the early stage of response inhibition^8^,^12^; the N2 latency would reflect the speed of this process^13^. A smaller and delayed NoGo-N2 component was found in the group of people with impaired inhibitory functioning^14^,^15^,^16^. Second, the central P3, a positive wave in the 300–600 ms time range, is larger in the NoGo than in the Go condition, which represents a later stage of the response inhibitory process^17^. The larger NoGo-P3 was thought to directly reflect conflict resolution through top-down inhibition processing^18^,^19^; its amplitude was related to cognitive demand and the attentional resources available for the task^20^. A smaller NoGo-P3 amplitude was found in the inhibition-impaired group^21^.

Additionally, the study of cognitive impairment due to high-altitude hypoxia in people who were born and raised in low-altitude areas and relocated in Tibet is increasingly important. Most prior research that has focused on local residents at high altitudes^1^,^22^ or individuals with acute exposure to high altitudes^23^,^24^ has found cognitive impairment caused by high altitude. However, the results from acute exposure or local residence at high altitude may not generalize to adult immigrants. This is because acute and chronic exposure to high altitudes affects cognition differently^25^, and local residents are different from low-altitude residents in terms of genetics and other physiological features^1^,^26^. The physiological and psychological changes in the immigrant group could reflect the effects of high altitude on cognition better than high-altitude residents or people with acute exposure to high altitudes. Study of cognitive changes in this population can provide a theoretical basis for the provision of cognitive training, prevention of cognitive impairment and other practical applications.

In the present study, we aimed to investigate the time course of the impact of chronic high-altitude exposure on the response inhibition process using ERPs in a Go/NoGo paradigm. In our study, the high-altitude group was determined by the same considerations used in our previous experiment^27^. We considered the participants to be more representative of the influence of high altitude because they had immigrated to a high-altitude area in adulthood and had acclimated to the environment while living there for three years. We predicted that the effects of chronic exposure to high altitude would be found on behaviour and ERP results in our study. For the behaviour result, we did not find any long-term high-altitude exposure study using the Go/NoGo task to discuss the influence of high-altitude hypoxia on response inhibition. Based on the neuroimaging evidence of the impact of high-altitude exposure on the human brain, we only could speculate that the effects of high altitude would be found in behaviour results (miss rates, commission errors, and reaction times). For the ERP results, the high-altitude effect on inhibition should be reflected in the N2 and P3 components; we predicted smaller and later NoGo-N2 components and smaller NoGo-P3 amplitudes would be found in the high-altitude group.

## Results

### Behavioural results

For the high-altitude and low-altitude groups, respectively, the average miss rate was 1.45 ± 3.76% (mean ± S.D.) and 0.60 ± 0.76%, and the commission errors (false alarms) were 17.40 ± 13.06% and 16.56 ± 10.92%. The average time for correct responses was 304 ± 63 ms for the high-altitude group and 305 ± 36 ms for the low-altitude group (Table 1). No significant differences were found in behavioural performance between these two groups (*p*s > 0.05). In the signal detection analysis, the difference between the two groups was not significant for *d’* [*t* (38) = −0.33, *p* = 0.75] and for *β* [*t* (38) = 0.98, *p* = 0.33].

### ERPs

**Figure 1:**
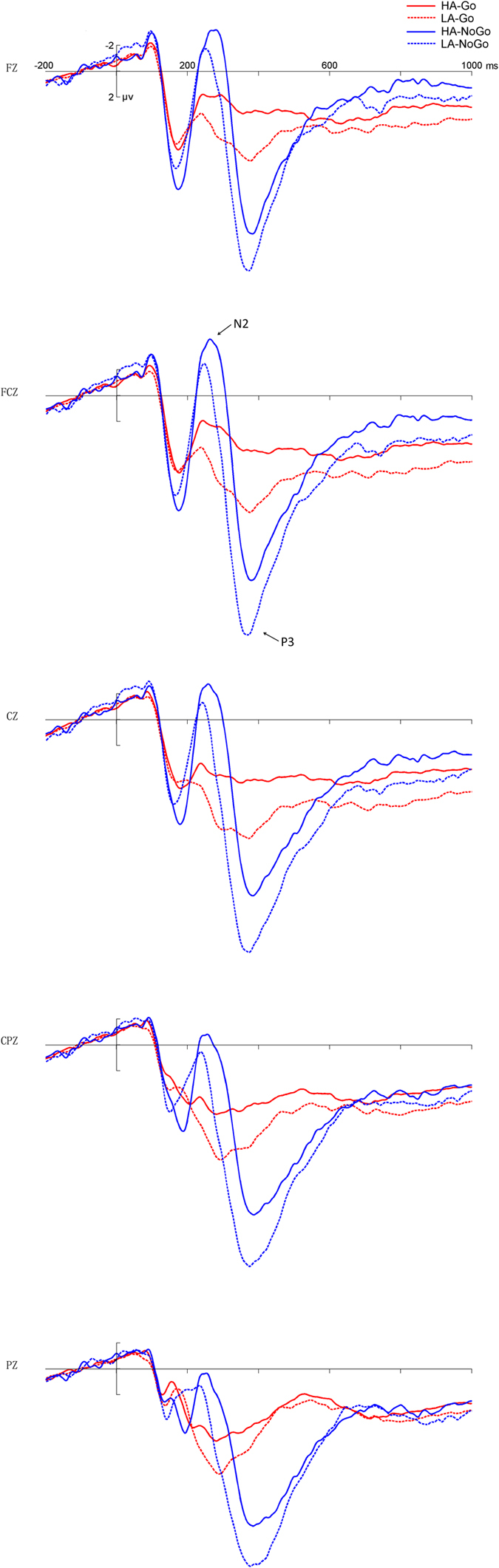
Grand average of ERP. The grand average of ERP elicited inthe low-altitude group (LA, dotted lines) and the high-altitude group (HA, solid lines) at the central sites (Fz, FCz, Cz, CPz Pz) in the Go (Go, red lines) and NoGo (NoGo, blue lines) conditions.

**Figure 2:**
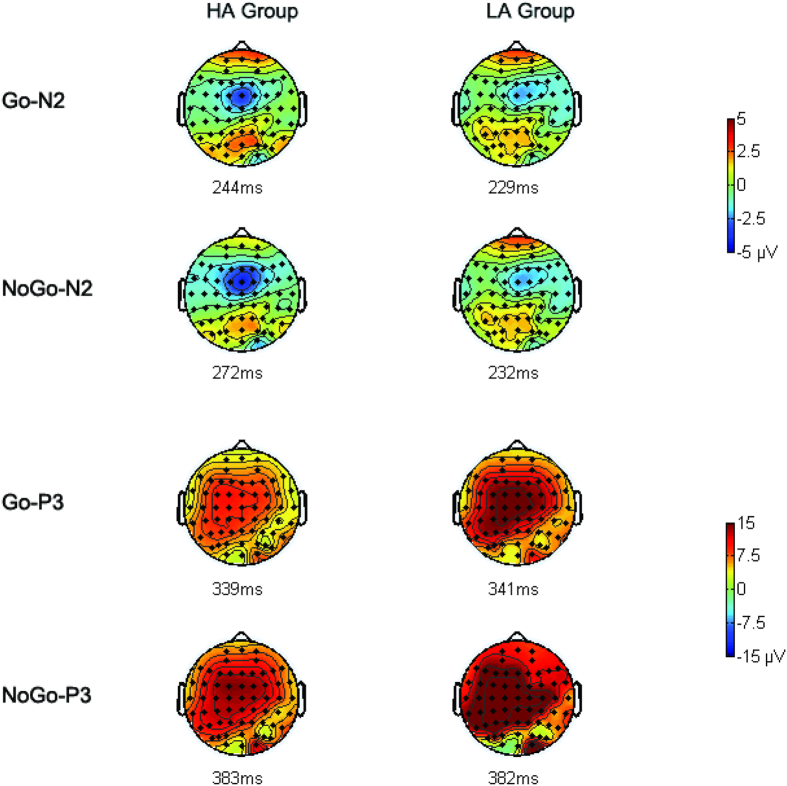
Topographical maps. The scalp distributions are time-locked to the peak amplitude of the N2 and P3 waves in the Go and NoGo conditions.

**N2**. With regard to the amplitude of the N2 component, the main effect of group was marginally significant [−2.22 ± 1.45 μV vs. 1.02 ± 1.15 μV; *F* (1,38) = 3.98, *p* = 0.053], with more negative N2 amplitude in the high-altitude group than in the low-altitude group (Fig. 1 and Fig. 2). The main effect of trial type was significant, with more negative N2 for NoGo stimuli than for Go stimuli in both groups [−3.47 ± 1.13 μV vs. 2.27 ± 0.72 μV; *F* (1,38) = 43.40, *p* < 0.001] (Fig. 1 and Fig. 2). The interaction between trial type and electrode site was significant [*F* (1,35) = 6.01, *p* = 0.005], and the trial type effect was significant at all three electrode sites [Fz: *t* (39) = 5.36, *p* < 0.001; FCz: *t* (39) = 6.21, *p* < 0.001; Cz: *t* (39) = 6.95, *p* < 0.001] (Table 2), with maximum effect at the Cz site. No other main effect or interaction was significant.

With regard to the latency of the N2 component, the interaction between trial type and group was significant [*F* (1,38) = 12.28, *p* < 0.001], with longer NoGo-N2 latency in the high-altitude group than in the low-altitude group [272.60 ± 4.47 ms vs. 232.67 ± 3.60 ms; *F* (1,38) = 48.41, *p* < 0.001]. The difference between the two groups was not significant in the Go trials [244.20 ± 6.64 ms vs. 229.33 ± 3.20 ms; *F* (1,38) = 0.71, *p* *>* 0.05] (Table 2).

**P3**. With regard to the amplitude of the P3 component, the main effect of trial type was significant, with a larger P3 amplitude for NoGo than for Go stimuli [16.23 ± 0.93 μV vs. 7.52 ± 0.63 μV; *F* (1,38) = 125.90, *p* < 0.001] (Fig. 1 and Fig. 2). The main effect of group was significant, with smaller P3 amplitude for the high-altitude group than for the low-altitude group [10.15 ± 1.50 μV vs. 13.60 ± 3.16 μV; *F* (1,38) = 7.01, *p* = 0.01] (Table 3). No other main effect or interaction was significant.

With regard to the P3 latency, the main effect of trial type was significant, with longer P3 latency for NoGo than for Go stimuli [383.64 ± 2.82 ms vs. 340.68 ± 2.01 ms; *F* (1,38) = 161.40, *p* < 0.001] (Table 3). No other main effect or interaction was significant.

### Source Localization

**Figure 3:**
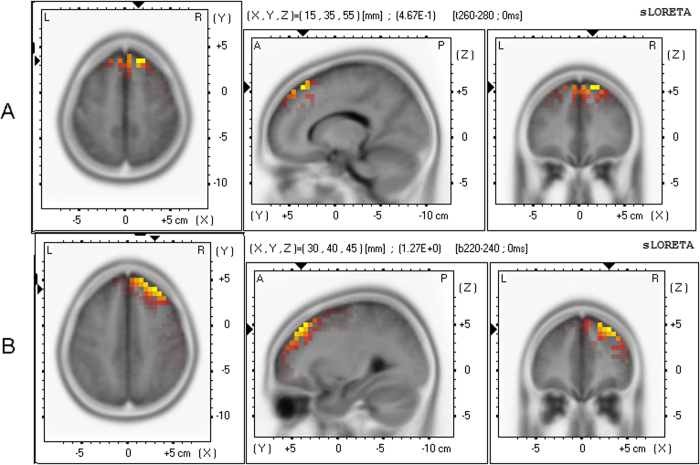
Source localization. The source localization of the surface NoGo-N2 amplitude sLORETA images showing the standardized current density maxima for the high-altitude group (**A**) and low-altitude group (**B**), as seen from the horizontal, sagittal, and coronal sections. Talairach coordinates (**X,Y,Z**) are indicated, the activity is colour-coded. Yellow colour indicates local maxima of the NoGo-N2 component is in the frontal Lobe (superior frontal gyrus and middle frontal gyrus) in the high and low altitude groups.

The sLORETA showed the strongest activation in the frontal lobe in both groups. Figure 3 shows the mean activation levels of the NoGo-N2 components in the brain regions of the two groups as plotted by standardized low resolution electromagnetic tomography analysis (sLORETA). The talairach coordinates were value = 4.67E-1, (X = 15 , Y = 35 , Z = 55) for the high-altitude group, and value = 1.27E + 0, (X = 30 , Y = 40 , Z = 45) for the low-altitude group.

## Discussion

Using a Go/NoGo task, our study investigated the neural mechanisms responsible for modulation of response inhibition in healthy young people after long-term exposure to high altitude. The results showed that a response inhibition effect was successfully elicited, as reflected by larger amplitudes of N2 and P3 in NoGo compared to Go trials in both groups. We focused our analyses on the differences between the high-altitude and low-altitude groups. The main results showed that a later NoGo-N2 was found in the high-altitude group compared to the low-altitude group, and the amplitude of N2 was larger and that of P3 smaller in the high-altitude group compared to the low-altitude group in both the Go and NoGo trials.

Inconsistent with our hypothesis, larger N2 amplitude was found for the high-altitude group than for the low-altitude group for both NoGo and Go conditions. According to the model-based object recognition system^28^,^29^, if the given object matches the explicit model, then familiarity is achieved. In this study, the processing of the Go stimulus is a top-down process which provides a recent context for the subsequent visual target. When the Go stimulus appears, the object matches the Go model, and when the NoGo stimulus appears, it mismatches with the model; the matching of the presented Go and/or NoGo stimuli with the internal representation of the Go responses was reflected in the N2 amplitude^30^,^31^. The result could be explained by generally increased neural activity in the matching step of information processing.

The amplitude of P3 was smaller for the high-altitude group than for the low-altitude group in both the NoGo and Go trials. According to the dissociable effects in more recent research, N2 and P3 are now thought to represent functionally separable processes in the Go/NoGo task^32^, NoGo-P3 amplitude was related to the late step of response inhibition^10^. However, we found that the P3 amplitude in both Go and NoGo trials were smaller in the high-altitude than in the low-altitude group. Beyond the field of response inhibition, the P3 component has generally been considered as a late stage of information processing, more specifically, as reflecting attentional resources being allocated to the task^7^,^33^. In our study, there was high cognitive demand in the high-altitude group, which would limit the attentional resources to resist the inhibitory control. The attentional resources declined in the high-altitude group compared to the low-altitude group, which produced smaller P3 in the high-altitude group than in the low-altitude group. Finally, in previous studies, decreased P3 amplitudes were found in aging adults (compared to young adults) and illness groups such as, Alzheimer’s disease patients, Parkinson’s disease patients and alcoholics with some degree of impaired inhibitory functioning^15^,^16^,^34^,^35^. In a high-altitude study that used an attention task, we also found decreased P3 amplitude in the high-altitude group compared to the low-altitude group^27^. Our previous results with regard to P3 amplitude suggest that long-term high-altitude exposure leads to diminished availability of attentional resources^36^.

More importantly, the present study found that the high-altitude group presented delayed NoGo-N2 latency compared to that of the low-altitude group. The peak latency of ERP component represents processing speed^13^,^37^. In our test, the processing of the Go stimulus provides a mental template for the subsequent visual target, the N2 component for NoGo stimuli reflects a mismatch between the current stimulus and a mental template^38^, and the NoGo-N2 latency results may reflect the mismatch processing speed. As in the mismatch condition, later NoGo-N2 latency was found in the high-altitude group compared to the low-altitude group, which suggested prolonged mismatch processing in the high-altitude group. According to previous studies^39^,^40^, the terms conflict and mismatch share some similarity and both implicate a process of comparison. When the information from a stimulus is transmitted into the brain, the information of the previous stimulus is retrieved and compared with the latter one. The information difference between the two stimuli indicates some kind of information mismatch or conflict^41^. According to a previous study^42^, the mismatch between the input stimulus and the current goal engendered cognitive conflict in the goal direct test. More specifically, in the Go/NoGo task, when the NoGo stimulus was compared with the Go stimulus, the comparison process led to a mismatch, and the mismatch between the NoGo stimuli and the current goal (intention to Go simuli) may have engendered conflict monitoring that is reflected in the NoGo-N2. In other words, the NoGo-N2 reflects a conflict monitoring process whenever it detects a mismatch. The longer latency of NoGo-N2 for the high-altitude group suggests that high-altitude subjects are slower in the timing of conflict monitoring, consistent with the hypothesis of cognitive deficits after long-term exposure to high altitudes^22^,^23^,^24^.

On the basis of the previous studies, the N2 component was located in the middle frontal, right inferior frontal, and ACC^10^,^11^ which correlated with conflict monitoring in the response inhibition process^30^,^43^. Using sLORETA, we localized the NoGo-N2 component in the superior frontal and middle frontal gyrus; maximal activation was found at the right side of the frontal area in our experiment (Fig. 3)^44^. The middle frontal cortex has been reported to be influenced by chronic hypoxia^5^,^6^, which may explain why delayed NoGo-N2 latency was found in the high-altitude group. Although the response inhibition effect was reflected in the amplitude of NoGo-N2 in most of the previous studies^10^,^45^, the latency was also a very important indicator of the ERP component. It is a sensitive index of the timing of information processing during visual perception, and the N2 latency reflected the slowdown in the processing speed of response inhibition in the conflict-monitoring stage.

The group differences were not significant in terms of behaviour results. It is an interesting question as to how the high-altitude group managed to preserve task performance. Previous acute exposure studies using different cognitive tasks have found increased reaction time in high-altitude areas^46^,^47^,^48^, but the opposite result was found with prolonged exposure to high altitude^22^,^49^. This finding may have occurred because acute and chronic exposure to high altitudes affect cognition differently^25^. The disappearance of the effects on behaviour after prolonged exposure may result from adaptation supported by a compensatory mechanism, which was also found in our previous study^27^. After the stimulus appeared, delayed NoGo-N2 latency was found in the high-altitude group than the low-altitude group, which reflected the group difference on the processing time in the early stage of response inhibition. However, N2 amplitude in the high-altitude group was enhanced in both the Go and NoGo trials. As the amplitude of ERP component reflects the neural activity level, the increased N2 amplitude in high-altitude group suggests that the high-altitude group, in comparison, engaged a higher level of neural activity to finish the same task. Therefore, the slower processing at the early stage did not slacken the late processing stage or affect the behavioral results. The group differences were not significant on P3 latency and the behavioural result. With a more difficult task, the behavioural effects may be more obvious. From another aspect, the disappearance of effect at the behavioural level may also be due to lower sensitivity of the behavioural measure used.

The primary limitation of the present research was that the climatic or cultural effects of high altitude should also be considered. Although complete physical adaptation to 3,600 m occurs after 40 days^25^, and three years’ exposure to high altitude is sufficient for acclimation, there may still be climatic or cultural effects; thus, the results should be interpreted cautiously.

In conclusion, the present findings reveal that chronic high-altitude exposure affected response inhibition, and specifically, processing speed at the conflict-monitoring stage, as indicated by the later NoGo-N2 latency found in the high-altitude group. A general high-altitude effect was also found in terms of matching step of information processing and attentional resources for both the Go and NoGo conditions, as indicated by the larger N2 and smaller P3 components elicited in the high-altitude group compared to the low-altitude group. Because there is no previous ERP study on response inhibition after long-term exposure to high altitude, these findings make a valuable contribution to the basic science of altitude effects on cognition.

## Methods

### Participants

Forty healthy young college students from the Han ethnic group, aged 21–24 years old, took part in this experiment. All participants signed an informed consent form before the experiment. The experiment was conducted in accordance with the Declaration of Helsinki and was approved by the Ethics Committee of the Institute of Psychology, Chinese Academy of Sciences. All participants were right-handed and had normal or corrected-to-normal vision. All participants had been born and raised in a low-altitude location (<1000 m). The twenty participants in the high-altitude group (10 male, 21.78 ± 1.41 years) had lived at high altitude (3650 m) for three years, and the twenty participants in the low-altitude group (10 male, 22.75 ± 1.08 years) had never been to a high-altitude area.

### Procedure

**Figure 4:**
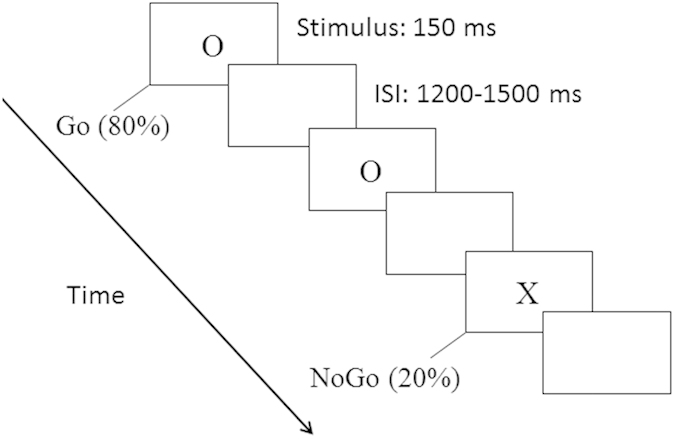
Materials and procedure. The procedure of the experimental paradigm, ‘O’ for Go trials, and ‘X’ for NoGo trials.

The visual stimuli for the Go/NoGo task were the capital letters ‘O’ and ‘X’. White visual stimuli were presented against a black background in the centre of a computer screen (AOC 17-in. LCD monitor), with a visual angle of approximately 2.6° vertically and 1.8° horizontally. One of the two letters was presented in a single trial, and either a response (Go) or the with holding of a response (NoGo) was required of the participant. The association between letters and trial type was counter balanced between the two blocks, with the letter ‘O’ serving as a Go stimulus in one block and as a NoGo stimulus in the other block. Each trial began with a Go or a NoGo stimulus lasting for 150 ms, followed by a black screen. The inter-stimulus interval (ISI) range was 1200–1500 ms (Fig. 4).

Participants were tested individually in a dimly lit, sound-attenuated room. After 20 practice trials, two experimental blocks of 240 trials in each block were completed, with 192 Go (80%) and 48 NoGo (20%) trials per block. Most of the trials were Go trials; thus, when the NoGo stimulus appeared, participants had to control their impulse to respond, inducing the impulse inhibition process. Behavioural data were collected and the stimuli were presentated using the E-prime software system (Version 1.1, Psychology Software Tools, Inc., Pittsburgh, PA). Participants were instructed to press ‘M’ with the right hand on a computer keyboard when a Go stimulus occurred and to give no response to a NoGo stimulus. Speed and accuracy were equally emphasized.

### EEG recording

Electroencephalography (EEG) data were recorded from 64 scalp sites (10/20 system), using Ag/AgCl electrodes mounted in an elastic cap (Neuroscan Inc.). The physical reference electrode was approximately 2 cm posterior to CZ. Electrode impedances were kept below 5 kΩ. Vertical and horizontal electrooculogram (EOG) data were recorded from above and below the left eye and from the outer canthi of both eyes, respectively. EEG and EOG were continuously recorded at a sampling rate of 500 Hz, applying a filter bandwidth of 0.05–100 Hz.

### Data analysis

Data were analyzed with SPSS (SPSS, Inc., Chicago) for Windows. P < 0.05 were considered statistically significant. The reaction time, the miss rate, and the commission errors (false alarm) from both groups were subjected to independent t-tests comparing the high-altitude and low-altitude groups. Signal detection analysis was also used to analyze the behavioural data. The sensitivity index (*d’*) and response bias index (*β*) according to the signal detection theory were calculated^50^. The hit and false alarm rates were transformed into z scores. Trial type was a within subjects factor and group was a between subjects factor. Independent-samples t-test was used to compare the high-altitude and low-altitude groups.

The EEG data were re-referenced to the average of left and right mastoid (M1 and M2). Ocular artifacts were removed from the EEG signal using a regression procedure implemented with Neuroscan software^51^. The ERP data were digitally filtered with a 40 Hz low pass. Continuous EEG data was segmented into stimulus-locked (−200 ms to 1000 ms) ERP segments, including a 200 ms pre-stimulus baseline. Trials with various artifacts were rejected, with a criterion of ±75 μV. Waveforms averages for each individual subject within each condition were calculated.

Peak amplitudes and peak latencies were used for statistical analyses of the N2 and P3 components. The time windows for the peak detection of N2 and P3 components were 200–300 ms and 250–450 ms, respectively. Electrode sites for analysis were chosen based on the scalp distributions of the current data and previous research demonstrating that the N2 is focal over the fronto-medial locations and P3 is over the midline electrodes^7^,^30^. Fz, FCz, and Cz were selected for N2; Fz, FCz, Cz, CPz, and Pz were selected for the P3 data analysis^30^,^52^,^53^. The amplitude and latency of N2 were subjected to a mixed-model ANOVA, respectively. The ANOVA factors for the N2 component included trial type (two levels: Go and NoGo), electrode sides (three levels: Fz, FCz, Cz) as within-subject factors, and altitude group (two levels: high altitude and low altitude) as between-subject factor. The amplitude and latency of P3 were also subjected to a mixed-model ANOVA, respectively. The ANOVA factors included trial type (two levels: Go and NoGo), electrode sides (five levels: Fz, FCz, Cz, CPz, Pz) as within-subject factors, and altitude group (two levels: high altitude and low altitude) as between-subject factor. The Greenhouse–Geisser correction was used to compensate for sphericity violations. Simple effect analyses were conducted to explore interaction effects.

Source localization of NoGo-N2 amplitude values was plotted using the sLORETA^44^. The sLORETA is an efficient tool for functional mapping and source localizing; it is consistent with physiology localization. Fifty-eight electrodes (the M1, M2, two VEOG electrodes, and two HEOG electrodes were excluded from the 64 electrodes) were used for analysis. A transformation matrix was created using the electrode coordinates. The averaged waveforms in the NoGo-N2 time range were converted and saved into ASCII values for both the high-altitude and low-altitude groups. sLORETA images were constructed based on the high-altitude and low-altitude group data, respectively. The version of sLORETA employed in our study was made available at http://www.unizh.ch/keyinst/NewLORETA/LORETA01.htm.

## Additional Information

**How to cite this article**: Ma, H. *et al.* Long-Term Exposure to High Altitude Affects Response Inhibition in the Conflict-monitoring Stage. *Sci. Rep.* **5**, 13701; doi: 10.1038/srep13701 (2015).

## Figures and Tables

**Table 1:** Mean reaction time, miss rate, commission errors and standard errors for high-altitude group (HA) and low-altitude group (LA).

	RT (ms)	miss rate (%)	commission errors (%)
HA	304 (63)	1.45 (3.76)	17.40 (13.06)
LA	305 (36)	0.60 (0.76)	16.56 (10.92)

**Table 2:** N2 mean amplitudes in μV (SE) and mean latencies in ms (SE) during the Go and NoGo conditions, separated between low-altitude (LA) and high-altitude (HA), at three midline electrodes (Fz, FCz, Cz).

	Amplitudes				Latencies			
	Go		NoGo		Go		NoGo	
	LA	HA	LA	HA	LA	HA	LA	HA
Fz	2.73(1.08)	0.72(1.12)	−0.38(1.18)	−5.74(1.88)	226.60(3.73)	248.60(7.74)	230.60(4.46)	276.80(4.82)
FCz	3.04(0.99)	1.05(1.08)	−1.70(1.48)	−6.75(1.88)	230.80(4.55)	243.80(7.35)	233.20(4.32)	270.40(4.23)
Cz	3.82(0.94)	2.26(1.02)	−1.37(1.23)	−4.89(1.72)	230.60(4.74)	240.20(8.37)	234.20(4.43)	270.60(5.19)

**Table 3:** P3 mean amplitudes in μV (SE) and mean latencies in ms (SE) during the Go and NoGo conditions, separated by low-altitude (LA) and high-altitude (HA) group, at five midline electrodes (Fz, FCz, Cz, CPz, Pz).

	Amplitudes				Latencies			
	Go		NoGo		Go		NoGo	
	LA	HA	LA	HA	LA	HA	LA	HA
Fz	7.62(0.63)	4.45(1.23)	16.37(1.58)	14.07(1.16)	377.00(4.04)	364.20(7.09)	378.50(5.19)	379.90(3.78)
FCz	9.82(0.74)	5.12(1.38)	19.56(1.70)	15.76(1.20)	373.50(5.68)	371.90(7.37)	371.70(4.68)	378.20(4.27)
Cz	10.24(0.67)	5.62(1.26)	19.11(1.57)	14.92(1.22)	374.20(6.27)	371.80(4.64)	378.60(4.73)	379.10(4.30)
CPz	9.77(0.73)	6.69(0.85)	17.55(1.55)	14.38(1.21)	295.30(4.73)	300.00(6.87)	395.30(6.02)	386.60(4.50)
Pz	9.17(0.73)	6.68(0.60)	16.79(1.09)	13.82(1.17)	286.90(5.00)	292.00(6.68)	395.70(5.69)	392.80(6.37)
